# Decoding *Akkermansia muciniphila* Effector Biology: From Microbial Molecules to Host Outcomes

**DOI:** 10.1002/mbo3.70369

**Published:** 2026-07-29

**Authors:** Amir Arsalan Ghahari, Ainur Sadykova, Salim Davlatov, Mukaddas Xusanova, Gulrukh Indiaminova, Furkat Sobirov, Mirzamakhmud Shadmanov, Umidjon Maxamatov, Patkhiddin Nishonov, Seyed Davar Siadat

**Affiliations:** ^1^ Department of Mycobacteriology and Pulmonary Research Pasteur Institute of Iran Tehran Iran; ^2^ Microbiology Research Center (MRC) Pasteur Institute of Iran Tehran Iran; ^3^ Department of Infectious and Tropical Diseases S.D. Asfendiyarov Kazakh National Medical University Almaty Kazakhstan; ^4^ Department of Faculty and Hospital Surgery Bukhara State Medical Institute named after Abu Ali ibn Sino Bukhara Republic of Uzbekistan; ^5^ Department of Anatomy Alfraganus University Tashkent Republic of Uzbekistan; ^6^ Department of Obstetrics and Gynaecology No. 3 Samarkand State Medical University Samarkand Republic of Uzbekistan; ^7^ Department of Obstetrics and Gynecology Tashkent State Medical University Tashkent Republic of Uzbekistan; ^8^ Department of Urology Andijan State Medical Institute Andijan Republic of Uzbekistan; ^9^ Department of Nutrition, Child and Adolescent Hygiene Fergana Medical Institute Public of health Fergana Uzbekistan; ^10^ Department of Practical French Language Studies Uzbekistan State World Language University Tashkent Uzbekistan

**Keywords:** *Akkermansia muciniphila*, Amuc_1100 (pilus‐associated signaling, PAS), effector‐centered framework, extracellular vesicles (EVs), microbiota–gut–brain axis, neuroinflammation, outer membrane vesicles (OMVs)

## Abstract

*Akkermansia muciniphila* is increasingly linked to host metabolic, immune, and neurobehavioral phenotypes, yet taxon‐level associations are often inconsistent across studies and disease contexts. This review advances an effector‐centered framework to explain how *A. muciniphila* acts through host‐facing molecules, including outer membrane and secreted proteins such as Amuc_1100 (pilus‐associated signaling, PAS) and P9, extracellular vesicles (EVs) and outer membrane vesicles (OMVs), and shed cell‐envelope fragments and lipids. We synthesize evidence spanning barrier biology, immunometabolic regulation, infection and inflammatory injury, cancer immunology, and microbiota–gut–brain axis research. Across models, effectors modulate the mucus and epithelial barrier to limit translocation and dampen Toll‐like receptor (TLR) signaling, but mucus‐active enzymes or antigenic epitopes may also create liabilities in susceptible hosts. Defined effectors can reproduce key whole‐cell effects: in high‐fat diet (HFD)/carbon tetrachloride (CCl4) liver injury, vesicle preparations matched or exceeded pasteurized bacteria; Amuc_1100 maps to immune reprogramming and barrier signaling; and P9 links microbial cues to glucagon‐like peptide 1 (GLP‐1) release. Key gaps include physiological dose realism, equivalence across colony‐forming units (CFU) and protein or vesicle doses, strain and culture‐condition dependence of effector expression, and limited human data for brain‐relevant endpoints. Effector‐defined preparations and engineered delivery systems may improve standardization and safety, but translation will require rigorous characterization, dose‐response validation, and mechanism‐linked biomarkers.

## Graphical abstract

Schematic overview of the proposed gut–brain axis mechanisms linking Akkermansia muciniphila to host outcomes. A. muciniphila‐derived signals, including SCFAs and P9/GLP‐1‐related endocrine pathways, may influence epithelial barrier integrity, inflammatory responses, systemic immune signaling, and neural communication through the enteric nervous system, vagus nerve, and CNS‐associated pathways. These interactions provide a conceptual framework for understanding how A. muciniphila may affect metabolic, immune, and brain‐relevant processes.

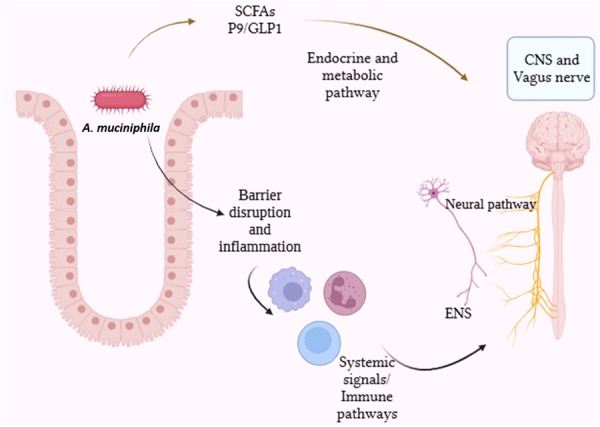

**Abbreviations**
5‐HT: 5‐hydroxytryptamine (serotonin)
ALPK1: alpha‐protein kinase 1
AMPK: adenosine monophosphate‐activated protein kinase
BBB: blood–brain barrier
BDNF: brain‐derived neurotrophic factor
CCl4: carbon tetrachloride
CFU: colony‐forming units
CHOP: C/EBP homologous protein (DDIT3)
CREB1: cAMP response element‐binding protein 1
DSS: dextran sulfate sodium
ED50: half‐maximal effective dose
ER: endoplasmic reticulum
EVs: extracellular vesicles
FITC: fluorescein isothiocyanate
FXR: farnesoid X receptor
GCG: proglucagon gene (glucagon precursor)
GI: gastrointestinal
GLP‐1: glucagon‐like peptide 1
GRP78: glucose‐regulated protein 78 (HSPA5/BiP)
HFD: high‐fat diet
HSL: hormone‐sensitive lipase
IECs: intestinal epithelial cells
IHC: immunohistochemistry
IL: interleukin
JAK–STAT: Janus kinase–signal transducer and activator of transcription
LAL: limulus amebocyte lysate
LOS: lipooligosaccharide
LPS: lipopolysaccharide
MAFLD: metabolic dysfunction‐associated fatty liver disease
MAPK: mitogen‐activated protein kinase
MASH: metabolic dysfunction‐associated steatohepatitis
MUC2: mucin 2
NF‐κB: nuclear factor kappa B
NOD1/2: nucleotide‐binding oligomerization domain‐containing protein 1/2
NTA: nanoparticle tracking analysis
OGTT: oral glucose tolerance test
OMVs: outer membrane vesicles
PAS: pilus‐associated signaling (Amuc_1100 context)
PCSK1: proprotein convertase subtilisin/kexin type 1
PD‐L1: programmed death‐ligand 1 (CD274)
PPAR: peroxisome proliferator‐activated receptor
PRRs: pattern‐recognition receptors
qPCR: quantitative polymerase chain reaction
ROS: reactive oxygen species
SCFAs: short‐chain fatty acids
SERT: serotonin transporter (SLC6A4)
TEER: transepithelial electrical resistance
TEM: transmission electron microscopy
TGR5: Takeda G protein‐coupled receptor 5 (GPBAR1)
TJ: tight junction
TLR: Toll‐like receptor
Tph1: tryptophan hydroxylase 1
Tregs: regulatory T cells
WB: Western blot
XBP1s: spliced X‐box binding protein 1
ZO‐1: zonula occludens‐1 (TJP1)

## 1 Introduction

*A. muciniphila* has emerged as a prominent mucosa‐associated bacterium repeatedly linked to host metabolic health, barrier integrity, and immunoregulation (FakhriRavari and Nguyen 2025). Yet, as the field has expanded into cancer, infection, and neuroscience‐adjacent outcomes, a central tension has become unavoidable: the same taxon can correlate with benefit in one context and with disease signatures in another, and even within a single disorder, the reported direction of association may conflict across cohorts (Mruk‐Mazurkiewicz et al. 2024). Reviews focused on neuropsychological and stress‐related outcomes emphasize this heterogeneity and warn against a simplistic “more is better” narrative, highlighting the influence of diet, medications, geography, and host state on both abundance and function (Lei et al. 2023; Misera et al. 2024). The practical consequence is that taxonomy alone is an imprecise explanatory unit for mechanism or translation. A more mechanistically faithful approach is to shift from microbe‐centered language to an effector‐centered framework. The host interfaces with molecules and structures that are produced, displayed, secreted, packaged, or shed by *A. muciniphila*, rather than with a name in a sequencing table. In this view, candidate effectors include surface and secreted proteins such as Amuc_1100 (also discussed as the pilus‐associated signaling, PAS, protein), secreted proteins such as P9, EVs and OMVs, and cell‐envelope fragments including peptidoglycan‐derived muropeptides and immunomodulatory membrane lipids (E.‐J. Kang et al. 2024). Segers and de Vos synthesize this logic by treating *A. muciniphila* as a platform of host‐active molecules, while repeatedly stressing that physiologic dose realism and accurate localization of activity remain major unresolved issues (Segers and de Vos 2023). Critically speaking, the “effector lens” also helps explain why experimental conclusions depend strongly on culture conditions, processing, and delivery format. Transcriptomic work shows that mucin availability can reprogram extracellular protein expression, including the induction of Amuc_1100 under mucin‐depleted growth, implying that what is administered in different studies may not be molecularly equivalent even if the same strain name is used (Sulaiman et al. 2025). Moreover, disease models increasingly demonstrate that purified components can recapitulate or even outperform whole‐cell interventions, as reported for vesicle preparations and for protein effectors that map to specific signaling nodes, including innate immune receptors and endocrine relays (Mo et al. 2024; Y.‐d. Wang et al. 2025; Zhao et al. 2025). This review therefore decodes *A. muciniphila* biology from microbial molecules to host outcomes, with particular attention to (i) how effector classes are generated and what the host is likely to encounter in vivo, (ii) the mucus and epithelial barrier as a proximal hub that gates systemic consequences, and (iii) downstream immune, metabolic‐endocrine, and neuroactive pathways relevant to the microbiota–gut–brain axis. We also highlight the translational inflection points and the key gaps that must be addressed before effector‐based *A. muciniphila* interventions can be credibly advanced for clinical neuroscience and related domains.

## 2 From Microbe to Molecule: What the Host Actually Encounters

In this review, EVs are used as the broader term for nanoscale membrane‐derived bacterial vesicles, while OMVs refers specifically to vesicles derived from the outer membrane of Gram‐negative bacteria such as *A. muciniphila*. *A. muciniphila* is often discussed as a single probiotic entity, yet the host rarely encounters it as a uniform, intact organism. At the intestinal surface, exposure is better framed as a mixture of viable and non‐viable cells plus host‐facing derivatives that accumulate during growth, stress, and host‐mediated clearance. These derivatives include surface structures, secreted proteins, EVs, OMVs, and cell‐envelope fragments (Noori et al. 2025; Zhao et al. 2025). This distinction matters because taxon‐level abundance can be a weak proxy for mechanism, particularly across heterogeneous clinical settings where diet, medications, and baseline mucosal state can shift both microbial ecology and microbial output (Mruk‐Mazurkiewicz et al. 2024; Ye and Cai 2025). Several lines of evidence support an exposure model rather than a strict colonization model: defined preparations such as vesicles, purified proteins, or conditioned supernatants can reproduce parts of the biological signal attributed to the whole organism (Jiang et al. 2025; Ye and Cai 2025). Conceptually, this implies that the relevant unit of action is not the species label in a sequencing table, but the molecular repertoire that reaches epithelial and immune sensing layers at the mucosal interface (Ye and Cai 2025). This framing also clarifies why upstream variables can dominate downstream conclusions. Culture context, especially mucin availability, can reprogram secretion‐associated programs and extracellular protein expression, implying that two preparations labeled “*A. muciniphila*” may encode different host‐facing payloads at administration (Mruk‐Mazurkiewicz et al. 2024). For neuroscience‐relevant translation, the key question therefore becomes which effector classes are generated under given conditions, how they are packaged or displayed, and which signals plausibly engage epithelial and immune pathways that can propagate into systemic physiology (Jiang et al. 2025; Zhao et al. 2025).

## 3 Effector Classes and Biogenesis: Building a Molecular Taxonomy

For conceptual clarity, host exposures can be organized hierarchically into: (i) whole‐cell preparations (live or pasteurized bacteria), which inherently contain multiple downstream components; (ii) subcellular fractions such as EVs and OMVs, which package selected microbial cargo; and (iii) defined molecular effectors including purified proteins, peptides, lipids, and peptidoglycan‐derived fragments. Importantly, higher‐order preparations may contain multiple lower‐level effectors simultaneously, which contributes to mechanistic overlap across studies. An effector‐centered view organizes *A. muciniphila* into a host‐facing repertoire of signals that differ in biogenesis, stability, and delivery constraints. Segers and de Vos group plausible mediators into four major classes: extracellular and surface‐exposed proteins, EVs or OMVs, metabolites, and cell‐envelope fragments. Importantly, this taxonomy is actionable because each class implies different expectations for gastrointestinal (GI) stability, receptor engagement, and standardization (Segers and de Vos 2023).

### 3.1 Surface and Secreted Proteins as Receptor‐Facing Cues

Among protein candidates, Amuc_1100, discussed as the PAS protein, is repeatedly positioned as a host‐interacting factor with plausible spatial presentation at the mucus interface. Functionally, Amuc_1100 has been linked to immunoregulatory outputs in inflammatory settings, supporting the concept that a defined protein can reproduce specific host programs without requiring long‐term colonization (Y.‐d. Wang et al. 2025; X. Zheng et al. 2023). A second axis is P9 (Amuc_1631), framed as a secreted factor that promotes GLP‐1 release, providing a mechanistic bridge from microbial signaling to endocrine physiology (Arukha et al. 2025; Yoon et al. 2021).

### 3.2 Vesicles as Packaged Multi‐Cargo Effectors

EVs and OMVs deliver proteins and lipids in nanoscale particles, creating multi‐signal exposure rather than single‐ligand stimulation. This packaging can protect cargo, concentrate signals, and facilitate interaction with epithelial cells across the mucus layer. Mechanistic studies often use component depletion logic to argue that vesicle‐associated cargo is not merely a byproduct but a functional delivery unit (Khalili et al. 2024; Noori et al. 2025).

### 3.3 Cell‐Envelope Fragments and Metabolites As Background Signaling Layers

Peptidoglycan‐derived fragments and immunoactive membrane lipids provide additional routes for innate sensing, while metabolites can modulate host physiology indirectly through redox, endocrine, or immune tone (Garcia‐Vello et al. 2024; Garcia‐Vello et al. 2022). However, mechanistic reviews emphasize that physiological exposure levels remain a central constraint, especially when in vitro dosing exceeds what is likely at the epithelial surface (Tables 1A, 1B, 1C) (Segers and de Vos 2023).

**Table 1A:** Effector entities of *Akkermansia muciniphila* and host‐related effects in preclinical studies.

Effector entity	Molecular class	Main host target/pathway	Representative outcomes	Key references
Amuc_1100 (PAS)	Outer membrane protein	TLR2; NF‐κB; AMPK; JAK–STAT/PD‐L1	Improved barrier integrity; reduced inflammation; enhanced CD8+ immune recruitment in cancer models; neuroactive marker modulation	Cheng et al. (2021); Cheng et al. (2022); Qu et al. (2023); Shi et al. (2022); Xu et al. (2024); Zhu et al. (2025)
P9 (Amuc_1631)	Secreted/functional protein	GLP‐1 secretion pathways; GCG/PCSK1 signaling	Improved glucose homeostasis; improved insulin sensitivity	Cani and Knauf (2021); Di et al. (2024); Yoon et al. (2021)
EVs/OMVs	EVs	AMPK/MAPK pathways; epithelial and immune signaling	Restoration of MUC2 expression; reduced ER stress; improved barrier function	Ashrafian et al. (2021); Raftar et al. (2022); Raftar et al. (2021); Zhao et al. (2025); T. Zheng et al. (2023)
Peptidoglycan fragments	Bacterial cell wall fragments	NOD1/NOD2; ALPK1–TIFA signaling	Induction of barrier genes; immune modulation	Garcia‐Vello et al. (2022); Martin‐Gallausiaux et al. (2022); Segers and de Vos (2023)
Membrane lipids/LOS	Lipid‐associated bacterial components	TLR signaling modulation	Context‐dependent immune regulation	Garcia‐Vello et al. (2024); Segers and de Vos (2023)

**Table 1B:** Formulation and processing modalities of *A. muciniphila*‐derived interventions in preclinical studies.

Formulation/processing modality	Effector source	Translational/application context	Representative outcomes across studies	Key references
Live *A. muciniphila*	Whole bacterium	Probiotic candidate (native strain administration)	Metabolic improvement; gut barrier modulation (context‐dependent across models)	Segers and de Vos (2023); Si et al. (2022)
Pasteurized *A. muciniphila*	Heat‐treated whole bacterium	Postbiotic‐like standardized preparation	Enhanced or retained metabolic and barrier effects in multiple models	Ashrafian et al. (2021); Raftar et al. (2021); Segers and de Vos (2023)
Recombinant Amuc_1100	Purified protein	Protein‐based postbiotic candidate	Barrier protection; immune modulation; anti‐inflammatory effects	Cheng et al. (2021); Cheng et al. (2022); Xu et al. (2024)
Purified P9 (Amuc_1631)	Recombinant/purified protein	Engineered metabolic therapeutic candidate	Enhanced insulin sensitivity and glucose regulation	Cani and Knauf (2021); Di et al. (2024)
EV/OMV‐based preparations	Bacterial EVs	Vesicle‐based postbiotic platform	Barrier restoration; mucin regulation; ER stress reduction	Raftar et al. (2021); Raftar et al. (2022); Zheng et al. (2023)
Lipid‐/fragment‐derived mimetics	Synthetic or purified bacterial components	Experimental immunomodulatory agents	Immune modulation; pathway‐specific signaling tuning	Garcia‐Vello et al. (2022); Garcia‐Vello et al. (2024)

**Table 1C:** *Akkermansia muciniphila*–based interventions in human studies.

Population	Product/strain & modality	Dose	Duration	Endpoints assessed	Outcomes	Safety/adverse events	Key reference
Overweight/obese adults (proof‐of‐concept RCT)	Pasteurized *A. muciniphila* (oral supplement; next‐generation probiotic)	10^10^ cells/day	3 months	Insulin sensitivity, weight, lipid profile, inflammatory markers, safety	Improved insulin sensitivity; modest reduction in body weight; improved metabolic markers	Well tolerated; no serious adverse events reported	Depommier et al. (2019)
Overweight/obese adults (same cohort, mechanistic analysis)	Pasteurized *A. muciniphila*	10^10^ cells/day	3 months	Gut barrier integrity, metabolic endotoxemia, inflammatory markers	Improved gut barrier markers; reduced metabolic inflammation signals	Safe and well tolerated	Depommier et al. (2019); FakhriRavari and Nguyen (2025)
Metabolic disease cohorts (systematic/clinical synthesis)	Live and pasteurized *A. muciniphila* (various formulations)	Variable (strain‐dependent; CFU or cells)	Variable (weeks–months)	Glycemic control, insulin resistance, lipid profile	Trends toward improved metabolic parameters across studies; heterogeneity in magnitude	Generally safe in reported short‐term studies	He et al. (2025); Mierlan et al. (2025)
Clinical trial registry (ongoing/early evidence)	Live *A. muciniphila* (NCT02637115)	~10^10^ cells/day (reported)	~3 months	Metabolic endpoints, liver enzymes, safety	Reported improvements in insulin sensitivity and liver enzymes (pilot‐level evidence)	No major safety concerns reported in summaries	NCT02637115

## 4 Context Dependence and Standardization: Why Results Diverge Across Studies

*A. muciniphila* is not a fixed exposure. Across studies, culturing conditions, processing, delivery format, and dosing conventions collectively reshape the effector payload that reaches the host interface (Si et al. 2022). In an effector‐centered framework, these variables are not technical noise; they are mechanistic modifiers that can change both magnitude and direction of reported outcomes, even when the same strain name is used (Han et al. 2025; E.‐J. Kang et al. 2024). Emerging evidence suggests that *A. muciniphila* is not functionally uniform but instead consists of multiple phylogroups or clades with potentially distinct metabolic and immunomodulatory properties. Recent studies indicate that extracellular vesicle composition, colonization behavior, and host immune interactions may vary across clades, supporting the possibility that effector repertoires are strain‐dependent rather than species‐universal (L. Mei et al. 2025). Although comparative effector mapping remains incomplete, strain‐level heterogeneity likely contributes to inconsistent findings across cohorts and experimental systems.

### 4.1 Growth Conditions Program the Effector Profile

Several upstream variables may reshape the effector profile of *A. muciniphila*. Mucin availability, nutrient composition, oxygen exposure, and growth phase can alter extracellular protein expression and vesicle cargo composition, while processing methods such as pasteurization may selectively preserve or attenuate specific host‐facing signals. Collectively, these variables may substantially influence biological activity and help explain inconsistent outcomes across studies using nominally similar bacterial preparations (Segers and de Vos 2023; Smith et al. 2023). Transcriptomic comparisons indicate that mucin availability can rewire extracellular and secretion‐associated programs, including increased Amuc_1100 expression under mucin depletion, potentially reflecting adaptive enhancement of host‐interface signaling during nutrient stress. However, whether this response is conserved across all strains or phylogroups remains unclear (Geerlings et al. 2024; Liu et al. 2021; X. Zheng et al. 2023).

### 4.2 Processing and Delivery Reweight Bioactivity

Comparative work suggests that potency is preparation‐dependent. In a HFD plus CCl4 liver‐injury model, live bacteria and EVs produced more consistent improvements than pasteurized cells, supporting the idea that heat processing can blunt or shift key host‐facing signals (Raftar et al. 2021). Conversely, engineered delivery can preserve function for defined effectors, as shown by heterologous secretion of P9 retaining GLP‐1 stimulatory activity in enteroendocrine models (Yoon et al. 2021).

### 4.3 Exposure Equivalence Remains the Core Comparability Gap

A persistent limitation is that “dose” is rarely comparable across formats, because CFU, purified‐protein mass, and vesicle‐protein mass are not interchangeable proxies for epithelial exposure. At minimum, studies should pair dose reporting with preparation‐specific characterization sufficient to interpret exposure: viability and verification of inactivation where relevant, standardized vesicle quantification beyond protein mass alone, and basic protein quality controls including purity and endotoxin burden (Davey et al. 2023; Lee et al. 2022). Without exposure‐matched dose–response designs, cross‐study synthesis risks over‐weighting the most concentrated preparation rather than the most physiologically relevant one (Table 2) (He et al. 2023; X. Zheng et al. 2023).

**Table 2:** Biomarker and endpoint matrix for mechanistic validation and gut–brain translation.

Mechanistic layer	Biomarkers/readouts	Mechanistic relevance	Level of evidence	Key refs
Product identity and exposure	Vesicle particle size/count, Amuc_1100/P9 quantification, endotoxin burden	Confirms exposure characterization and batch comparability	Preclinical	Davey et al. (2023); Segers and de Vos (2023); Zhao et al. (2025); T. Zheng et al. (2023)
Mucus and goblet‐cell function	MUC2, GRP78, CHOP, XBP1 splicing	Validates mucus restoration and ER‐stress modulation	Preclinical	Zhao et al. (2025); T. Zheng et al. (2023)
Tight junction integrity	Occludin, claudins, ZO‐1, TEER, FITC‐dextran assays	Assesses epithelial barrier integrity and permeability	Preclinical	Ashrafian et al. (2019); Jiang et al. (2024); Raftar et al. (2021); Shi et al. (2022)
Innate immune signaling	TLR/NF‐κB pathways, IL‐6, TNF, IL‐1β	Evaluates inflammatory and innate immune responses	Preclinical	Garcia‐Vello et al. (2024); Peña‐Cearra et al. (2024); Raftar et al. (2021); Segers and de Vos (2023)
Regulatory immune tone	IL‐10, macrophage polarization, Treg‐associated markers	Supports context‐dependent immune regulation	Preclinical	Anderson et al. (2024); Mulhall et al. (2020); Segers and de Vos (2023)
Endocrine‐metabolic signaling	GLP‐1, insulin, glucose tolerance, bile acid profiles	Validates endocrine relays linking gut to systemic physiology	Preclinical + limited human evidence	Di et al. (2024); He et al. (2025); Lin et al. (2025); Yoon et al. (2021)
Brain‐relevant outcomes	BDNF, CREB1, 5‐HT/SERT/Tph1, neuroinflammation markers, behavioral assays	Assesses indirect gut–brain axis signaling	Predominantly preclinical	Cheng et al. (2021); Cheng et al. (2022); Feng et al. (2025); Guo et al. (2024); E. J. Kang et al. (2024); Lei et al. (2023); Misera et al. (2024)

## 5 The Mucus Interface and Epithelial Barrier as the Proximal Hub

Across diverse disease models, the most reproducible early node is the mucus–epithelium interface. Before any downstream metabolic, immune, or neural effects can unfold, *A. muciniphila* effectors must reshape what intestinal epithelial cells (IECs) and goblet cells do: maintain mucin output, preserve TJ integrity, and limit luminal inflammatory translocation (Ashrafian et al. 2019; Mo et al. 2024).

### 5.1 Goblet‐Cell Programs and Mucin Restoration

A mechanistically clean example comes from the indomethacin goblet‐like cell injury model, where indomethacin reduced mucin 2 (MUC2) and activated endoplasmic reticulum (ER) stress. Purified *A. muciniphila* OMVs, as well as bacterial supernatant, restored MUC2 and reduced ER‐stress markers (e.g., GRP78, CHOP, and X‐box binding protein 1 splicing), while extracellular vesicle (EV) depleted supernatant failed to suppress the ER‐stress signature. This component swap strongly supports OMVs as an active barrier‐rescue unit rather than a vague “secreted factor” explanation (Zhao et al. 2025; T. Zheng et al. 2023).

### 5.2 Tight Junction (TJ) Maintenance and Permeability Control

**Figure 1:**
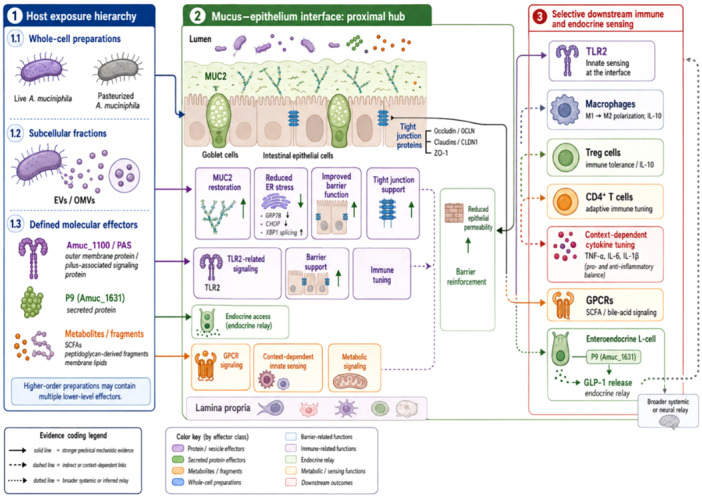
Effector‐centric actions of Akkermansia muciniphila at the mucus–epithelium interface. The schematic shows how host exposure to live or pasteurized A. muciniphila, EVs/OMVs, Amuc_1100/PAS, P9, and metabolites or fragments converges at the mucus–epithelium interface to support barrier integrity, immune tuning, and endocrine signaling. Solid arrows indicate stronger preclinical mechanistic evidence, dashed arrows indicate indirect or context‐dependent links, and dotted arrows indicate broader inferred relays. The figure is conceptual and effector‐mapped and does not imply that all effectors regulate all depicted pathways.

Barrier reinforcement is also reflected at the gene‐expression level in in vivo injury models. In the HFD and CCl4 liver‐injury study, the authors explicitly tracked colon histology and TJ transcripts (occludin, claudin‐1, claudin‐2) alongside hepatic inflammation and TLR programs, aligning improved tissue outcomes with a broader “gut‐liver axis” barrier logic (Jin et al. 2025). Complementary reviews summarize that *A. muciniphila* EVs and Amuc_1100 can increase TJ proteins through pathways frequently converging on AMP‐activated protein kinase (AMPK) signaling, and that EV preparations in inflammatory epithelial models can attenuate oxidative and mitogen‐activated protein kinase (MAPK) linked stress responses (Figure 1) (Jiang et al. 2024; Shi et al. 2022).

### 5.3 Why This Hub Matters for Brain Outcomes

For neuroscience‐relevant endpoints, the barrier is not merely a gut phenotype. Stress‐focused syntheses repeatedly link *A. muciniphila* interventions to restored goblet cells and MUC2 expression, lower systemic inflammatory tone, and downstream shifts in serotonergic and neurotrophic markers such as serotonin (5‐hydroxytryptamine, 5‐HT) and brain‐derived neurotrophic factor (BDNF) (E. J. Kang et al. 2024; S. Zhang et al. 2025). However, many studies still infer barrier repair from a limited set of markers, and dose equivalence across live cells, pasteurized preparations, proteins, and vesicles remains inconsistent (Mruk‐Mazurkiewicz et al. 2024). A priority gap is to pair standardized dosing with direct permeability assays and cell‐type‐specific readouts (goblet *vs.* absorptive IECs) to verify that barrier repair is truly causal rather than a correlated byproduct (Martin‐Gallausiaux et al. 2022).

## 6 Immune Reprogramming: From Pattern Recognition to Effector Responses

Across models, *A. muciniphila* effectors shape immunity in two tiers: upstream pattern‐recognition signaling (epithelial and myeloid sensing) and downstream reprogramming of effector functions (macrophage balance, cytotoxic T‐cell activity). Importantly, the signal is not simply “anti‐inflammatory.” It is better described as contextual tuning that reduces pathological inflammatory tone while preserving host defense capacity (Chi et al. 2025; Peña‐Cearra et al. 2024; Xie et al. 2025).

### 6.1 Pattern‐Recognition Receptors (PRRs) as the Entry Layer

Several studies converge on attenuated TLR linked inflammatory programs in injury states. In the HFD plus CCl4 liver‐injury model, live *A. muciniphila* and EVs reduced hepatic tlr‐5 and tlr‐9 expression versus injured controls, alongside lower pro‐inflammatory cytokines and improved histopathology. A recurring practical point is that processing changes immune‐facing biology: EVs and live bacteria tended to outperform pasteurized cells, suggesting that heat treatment can blunt or reweight key surface or vesicle‐associated signals even when the nominal “dose” is held constant (Ashrafian et al. 2021; Raftar et al. 2021). Mechanistically, Segers and de Vos (2023) emphasize that host dialog extends beyond a single outer membrane protein. They highlight peptidoglycan muropeptides that stimulate nucleotide‐binding oligomerization domain‐containing protein 1 and 2 (NOD1 and NOD2), and membrane lipids such as diacyl phosphatidylethanolamine that signal through a TLR2 and TLR1 heterodimer. Ornithine lipids are framed as candidates that bias toward interleukin‐10 (IL‐10) and anti‐inflammatory buffering rather than uniform suppression of innate sensing (Garcia‐Vello et al. 2022; Segers and de Vos 2023).

### 6.2 Macrophage Polarization and IL‐10 as a Controllable Axis

In *Porphyromonas gingivalis*–driven periodontitis, oral *A. muciniphila* or purified Amuc_1100 reduced alveolar bone loss and shifted macrophage balance toward an alternatively activated phenotype, with increased IL‐10 in gingival tissue and in bone marrow‐derived macrophage assays. This is conceptually important because it shows “postbiotic‐like” control: a defined outer membrane protein can reproduce an immunoregulatory phenotype without requiring durable colonization (Anderson et al. 2024; Mulhall et al. 2020).

### 6.3 Adaptive Immunity and Tumor Immune Escape

Immune effects also extend into adaptive circuits. In lung adenocarcinoma models, Amuc_1100 enhanced CD8‐positive T‐cell recruitment and cytotoxic mediators while reducing programmed death‐ligand 1 (PD‐L1), with results consistent with involvement of Janus kinase and signal transducer and activator of transcription (JAK–STAT) signaling. This widens the effector framework from barrier repair into immune‐checkpoint biology, while underscoring context dependence: what is beneficial in cancer immunity may not map cleanly onto chronic inflammation or stress phenotypes (Xu et al. 2024; Zhu et al. 2025). These immune effects should be interpreted in light of exposure non‐equivalence across formats, discussed in Section 4.3.

## 7 Metabolic‐Endocrine Relays Linking Gut Signals to Distal Organs

Building on the barrier‐first model (Section 5), endocrine relays offer a scalable route by which intestinal sensing can shape systemic physiology. In this view, *A. muciniphila* effectors act upstream of gut hormones, bile acid signaling, and inflammatory set points, enabling distal organ effects without requiring direct microbial translocation (Hagi et al. 2020; Niu et al. 2024).

### 7.1 Gut Hormone Signaling as an Effector Amplifier

The clearest endocrine route in this set is the GLP‐1 axis. Across diabetes‐focused syntheses, P9 is repeatedly positioned as a secreted effector that stimulates GLP‐1 release from enteroendocrine l‐cells, with downstream relevance for glucose homeostasis (Cani and Knauf 2021; Di et al. 2024; Yoon et al. 2021). Supporting the “portable effector” concept, one engineered‐delivery study reported that P9 secreted by *Lactococcus lactis* increased GLP‐1 output in an l‐cell model and upregulated proglucagon (GCG) and proprotein convertase 1 (PCSK1), implying that bioactivity can be delivered without administering live *A. muciniphila* (Di et al. 2024). Notably, endocrine claims still need tighter dose accounting, because most studies infer activity from supernatants or protein mass rather than quantifying active luminal exposure (Segers and de Vos 2023; Si et al. 2022; Yang et al. 2025).

### 7.2 Bile Acids, Lipid Handling, and Hepatic Signaling

Beyond GLP‐1, reviews converge on bile acid‐mediated signaling as a plausible bridge between gut ecology and systemic metabolism, including pathways involving receptors such as Takeda G protein‐coupled receptor 5 (TGR5) and inflammatory modulation (Gou et al. 2023; Lin et al. 2025; Song et al. 2023). Liver injury experiments reinforce that these relays are biologically meaningful: in a HFD plus CCl4 model, oral live bacteria and EVs reduced aminotransferases and improved histological injury scores while suppressing TLR associated inflammatory programs and restoring anti‐inflammatory regulators such as peroxisome proliferator‐activated receptors (PPARs) (Qu et al. 2023; Yan et al. 2023). The consistent pattern that EVs and live preparations outperformed pasteurized bacteria highlights a practical point for endocrine‐metabolic translation: processing reshapes the host‐exposed molecular profile and can shift the strength of downstream systemic effects (Tang et al. 2025; Wang et al. 2024; Y.‐d. Wang et al. 2025).

### 7.3 Redox and Inflammation as Endocrine‐Adjacent Multipliers

Endocrine relays are amplified or blunted by systemic inflammatory and oxidative states. Reviews emphasizing oxidative stress argue that Amuc_1100 and EVs can reduce reactive oxygen species (ROS) and lipid peroxidation while restoring antioxidant enzymes, thereby weakening the oxidative stress inflammation loop that accelerates insulin resistance and tissue injury (Liu et al. 2025; Ye and Cai 2025; Zeng et al. 2025). Nevertheless, many reports remain biomarker‐heavy, so the next step is integrative designs that jointly quantify hormone kinetics (e.g., GLP‐1 dynamics), receptor engagement, and tissue‐specific signaling in the same models.

## 8 Neuroactive Pathways and Gut–Brain Axis Translation

Downstream of epithelial and immune tuning (Sections 5), converging experimental and review evidence links *A. muciniphila* effectors to neuroactive readouts. Crucially, most proposed routes are indirect: effectors remodel the intestinal interface and immune tone, which can reshape endocrine and inflammatory signals that ultimately influence neural circuits (Guo et al. 2024; Sun et al. 2023). Importantly, the current evidence base for neuropsychiatric translation remains predominantly preclinical. Most mechanistic claims derive from rodent behavioral paradigms, inflammatory biomarkers, and neurotransmitter‐associated readouts rather than validated human clinical outcomes. Therefore, proposed gut–brain effects should be interpreted cautiously and viewed as hypothesis‐generating rather than clinically established mechanisms.

### 8.1 Stress and Depression‐Like Behavior: Serotonin‐Linked Signatures

**Figure 2:**
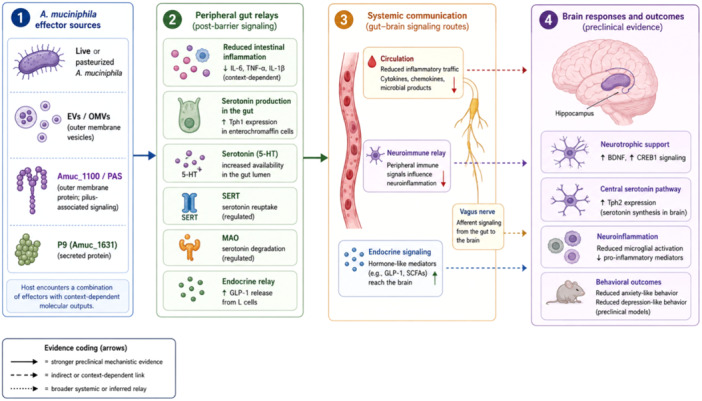
Effector‐linked gut–brain axis actions of Akkermansia muciniphila. The schematic illustrates how A. muciniphila effectors, including Amuc_1100/PAS, EVs/OMVs, and P9, may influence peripheral inflammatory, serotonergic, and endocrine pathways, which subsequently signal through circulatory, neuroimmune, and vagal routes to affect brain‐relevant outcomes. Proposed downstream effects include modulation of BDNF, CREB1, Tph2, neuroinflammation, and depression‐ or anxiety‐like behaviors. Most evidence supporting these mechanisms is currently derived from preclinical studies.

Misera et al. synthesize rodent stress paradigms in which *A. muciniphila* or the outer membrane protein Amuc_1100 is associated with improved depressive‐like readouts across standard behavioral assays (e.g., open field, tail suspension, forced swim, and sucrose preference) (Lei et al. 2023; Misera et al. 2024). Mechanistic summaries repeatedly converge on serotonin biology, including reduced gut serotonin transporter (SERT) expression and increased peripheral 5‐HT pools, alongside higher hippocampal BDNF and cAMP response element‐binding protein 1 (CREB1) and lower inflammatory cytokines in brain‐relevant tissues (Cheng et al. 2021; Ding et al. 2021). A modified Amuc_1100 variant with higher TLR2 affinity is discussed as increasing gut tryptophan hydroxylase 1 (Tph1), implying that effector structure can tune neurochemical outputs (Figure 2) (Cheng et al. 2022; Feng et al. 2025).

### 8.2 Neuroinflammation as the Relay Between Gut Effectors and the Brain

Feng J, et al. emphasize barrier disruption as a gatekeeper for systemic inflammation that can propagate neuroinflammatory programs (Feng et al. 2025). It highlights TLR2 to nuclear factor kappa B (NF‐κB) and adenosine monophosphate‐AMPK as recurring nodes through which Amuc_1100 and EVs may strengthen TJ programs and dampen inflammatory activation (Perry et al. 2025; Yang et al. 2022; Ye and Cai 2025). Nevertheless, direct central nervous system entry of effectors is rarely demonstrated, so “reduced inflammatory traffic” remains the more defensible bridge (Kurhaluk et al. 2025; Panzetta and Valdivia 2024).

### 8.3 Translation: Why Abundance Is Not a Biomarker by Default

Another recurring caution is heterogeneity: *Akkermansia* abundance trends differ by disorder and cohort. This argues against one‐size‐fits‐all augmentation and supports mechanism‐based stratification (host state, medication, diet, and strain‐level function) before clinical positioning (Ashrafian et al. 2021; Ashrafian et al. 2021; Feng et al. 2025; Mierlan et al. 2025; Yaghoubfar et al. 2020).

## 9 Discussion

An effector‐first interpretation of *A. muciniphila* is increasingly consistent: many host outcomes are transferable from the live microbe to defined products such as outer membrane proteins (notably Amuc_1100, also discussed as pilus‐associated signaling, PAS), secreted proteins (P9), and EVs or OMVs (He et al. 2025; Jian et al. 2023; E.‐J. Kang et al. 2024; L. Mei et al. 2025). Barrier‐first signaling recurs as the most consistent proximal node, with immune and endocrine relays accounting for many distal outcomes (Sections 5) (He et al. 2025; E.‐J. Kang et al. 2024; S. Y. Wang et al. 2025; Bingqi Zhang et al. 2025). Rather than assuming direct microbial access to peripheral organs, the evidence is more coherent when mucosal integrity gates inflammatory traffic and hormone‐like signals amplify downstream physiology (Feng et al. 2025; Y. Mei et al. 2025). Nevertheless, barrier reinforcement is better viewed as a common entry point than a complete explanation. Neurological and neuropsychological reviews repeatedly document heterogeneity in reported abundance changes across diseases and cohorts, implying that host state, medication exposure, geography, and strain‐level functional diversity can invert associations (Chikindas et al. 2026; Hong et al. 2025). Thus, “more *Akkermansia* is better” is not a defensible universal claim, particularly in vulnerable barrier contexts (Abolhassani et al. 2025; Feng et al. 2025; Ma et al. 2025). A major source of apparent contradiction is that “what the host actually encounters” varies substantially by preparation and growth context. For example, comparative in vivo work in a HFD plus CCl4 liver‐injury model shows EVs and live bacteria outperforming pasteurized bacteria across enzymes and histologic injury scores, consistent with the idea that heat processing can blunt or reweight key host‐facing signals while vesicle cargo remains potent under tested conditions (Ashrafian et al. 2021; Cheng et al. 2024; Raftar et al. 2022; Raftar et al. 2021). Complementing this, transcriptomic evidence that mucin availability reprograms extracellular protein expression, including strong induction of Amuc_1100 under mucin‐depleted growth, provides a mechanistic basis for lab‐to‐lab variability even when the same species name is used (Segers and de Vos 2023; Boqi Zhang et al. 2025; Zhang et al. 2022). The main translational bottleneck is exposure realism and dose equivalence across formats, including live cells, pasteurized preparations, proteins, and vesicles. This is why harmonized reporting and exposure‐matched dose–response designs are central priorities (Section 4.3) (Chen et al. 2023; Xu et al. 2024). Mechanistic specificity is improving but remains uneven by domain. Tumor models propose that Amuc_1100 can reduce immune escape through JAK–STAT signaling and PD‐L1 regulation, with increased CD8‐positive T‐cell recruitment and cytotoxicity (Shimizu et al. 2025; Xu et al. 2024; Zhu et al. 2024). In contrast, gut–brain axis work often links effectors to serotonin‐related markers, neurotrophic factors such as BDNF, and reduced neuroinflammation, but still rarely establishes a stepwise causal relay from gut sensing to defined neural circuit changes (Giambra et al. 2025; E. J. Kang et al. 2024; Lei et al. 2023). Priority next steps are clear: quantify effectors in administered products, establish dose‐equivalence frameworks for proteins and vesicles, pair receptor‐level perturbations with barrier and systemic mediators, and stratify by host baseline risk to move from promising associations toward predictable and safe interventions (Jiang et al. 2025; Wu et al. 2023).

### 9.1 Negative Aspects at the Effector Level: When Host‐Facing Molecules Backfire

Evidence for adverse outcomes is most defensible in this review only when it can be tied to specific effector classes that the host encounters.

#### 9.1.1 Mucus‐Degrading Enzymes and Mucinases

Tingler‐Engevik et al. described an extensive mucin‐degradation toolkit in *Akkermansia muciniphila*, including glycosyl hydrolases and mucinases (e.g., Amuc_1434, Amuc_1438, Amuc_0627, and OgpA). In fiber‐limited settings, these effectors could shift mucin turnover toward net inner‐mucus thinning, increasing epithelial proximity and inflammatory pattern‐recognition receptor signaling (Bakshani et al. 2025; Tingler and Engevik 2025). A key gap is that most studies do not directly quantify enzyme activity together with mucus penetrability under exposure‐matched designs.

#### 9.1.2 Antigenic Mimic Peptides

The strongest effector‐level autoimmune liability is molecular mimicry: a defined *A. muciniphila* peptide (P3, TTLSFYRPPFLRVRRPFYIIF) was reported to activate myelin‐reactive CD4‐positive T cells and aggravate experimental autoimmune encephalomyelitis under co‐immunization conditions (Ma et al. 2025). This mechanism is inherently effector‐centric because the causal unit is a sequence‐defined antigen. Nevertheless, in vivo expression levels and delivery routes remain insufficiently quantified.

#### 9.1.3 Immunogenic Surface or Vesicle‐Associated Antigens

Cruciani et al. reported intrathecal anti‐*A. muciniphila* immunoglobulin G responses in multiple sclerosis linked to cerebrospinal fluid immune features and lesion metrics in a limited imaging subset. Antibodies target antigens, implicating host exposure to surface, membrane, or vesicle‐associated molecules with central nervous system relevance (Cruciani et al. 2025; Vallino et al. 2020). The next major step is antigen mapping, because the specific molecular targets are still unknown. In conclusion, effector‐centric “risks” converge on three classes: mucin‐active enzymes that may compromise the mucus barrier under deprivation, mimic peptides that can engage autoreactive T‐cell circuits, and immunogenic surface or vesicle‐associated antigens that may be targeted within the cerebrospinal fluid compartment.

## 10 Clinical Implications

The effector‐centric view of *A. muciniphila* has direct clinical relevance because it offers a path to interventions that do not require durable colonization and may be easier to standardize than live biotherapeutics. Across reviews, pasteurized preparations and purified components such as Amuc_1100 (PAS) and EVs are repeatedly framed as more practical candidates for translation, particularly where safety, storage stability, and batch‐to‐batch consistency are essential (He et al. 2025; Zhao et al. 2024). This aligns with clinical experience in metabolic‐risk populations, where a randomized, double‐blind, placebo‐controlled pilot trial (NCT02637115) administering daily 10^10^ cells for 3 months reported improvements in insulin sensitivity and modest weight reduction, with favorable changes in liver enzymes in the trial summaries. Similarly, diabetes‐prevention focused synthesis highlights a proof‐of‐concept study in overweight volunteers using dailysupplementation for 3 months with reported tolerability and barrier‐related readouts, supporting feasibility rather than definitive efficacy (Depommier et al. 2019; FakhriRavari and Nguyen 2025; He et al. 2025; Ye and Cai 2025). Clinically, the most credible near‐term applications are in cardiometabolic and fatty‐liver risk states where endpoints are measurable, trials are practical, and mechanisms plausibly converge on barrier integrity and low‐grade inflammation (Mierlan et al. 2025). In contrast, neurological and stress‐related indications remain at an earlier stage. Although animal studies suggest shifts in serotonin‐related markers, neurotrophic factors such as BDNF, and behavioral readouts after *Akkermansia* or Amuc_1100 interventions, current reviews explicitly note that direct anti‐stress evidence is confined to experimental models (Abraham et al. 2025; Khalili et al. 2024). Therefore, clinical use for psychiatric or neurodegenerative outcomes should be considered investigational, not implementation‐ready. Key clinical constraints are product definition and exposure equivalence across preparations. Without harmonized characterization and clinically anchored dose–response evidence, comparisons across studies and products remain unreliable and may overstate efficacy. This makes standardized manufacturing metrics and mechanism‐linked biomarkers essential for responsible translation (Chikindas et al. 2026; L. Mei et al. 2025; Mierlan et al. 2025; Z. Zhang et al. 2025).

## 11 Conclusion

An effector‐focused framework clarifies why *A. muciniphila* can produce reproducible host benefits across diverse models while still yielding contradictory associations in human cohorts. The host does not respond to “*Akkermansia*” as a taxonomic label, but to a context‐shaped molecular output that includes surface proteins such as Amuc_1100 (PAS), secreted factors such as P9, EVs, and envelope‐derived immunomodulatory lipids and peptidoglycan fragments. Across studies, these effectors converge on a small set of proximal mechanisms, most consistently reinforcement of the mucus and epithelial barrier, followed by reprogramming of innate and adaptive immunity and propagation into metabolic, endocrine, and neuroactive pathways. Nevertheless, critical gaps remain: physiologic dose realism is rarely established, culture conditions and processing alter the effector profile, and standardized equivalence metrics are still missing, especially for vesicle and protein preparations. Clinically, the strongest translational footing currently lies in cardiometabolic and liver‐risk settings, whereas neurological applications require rigorous human validation with mechanism‐linked biomarkers.

## Author Contributions

**Amir Arsalan Ghahari:** conceptualization, writing – original draft, writing – review and editing. **Ainur Sadykova:** writing – original draft, writing – review and editing. **Salim Davlatov:** writing – original draft, writing – review and editing. **Mukaddas Xusanova:** writing – original draft, writing – review and editing. **Gulrukh Indiaminova:** writing – original draft, writing – review and editing. **Furkat Sobirov:** writing – original draft, writing – review and editing. **Mirzamakhmud Shadmanov:** writing – original draft, writing – review and editing. **Umidjon Maxamatov:** writing – original draft, writing – review and editing. **Patkhiddin Nishonov:** writing – original draft, writing – review and editing. **Seyed Davar Siadat:** writing – review and editing, supervision.

## Funding

The authors have nothing to report.

## Ethics Statement

The authors have nothing to report.

## Consent

The authors have nothing to report.

## Conflicts of Interest

The authors declare no conflicts of interest.

## Data Availability

Data sharing not applicable to this article as no datasets were generated or analyzed during the current study.
